# Derivative spectrophotometric method for simultaneous determination of clindamycin phosphate and tretinoin in pharmaceutical dosage forms

**DOI:** 10.1186/2008-2231-21-29

**Published:** 2013-04-10

**Authors:** Maliheh Barazandeh Tehrani, Melika Namadchian, Sedigheh Fadaye Vatan, Effat Souri

**Affiliations:** 1 Department of Medicinal Chemistry, Faculty of Pharmacy and Pharmaceutical Sciences Research Center, Tehran University of Medical Sciences, Tehran, Iran; 2 R & D Department, Kish Medipharm Company, No. 64, Kish free zone island, Iran

**Keywords:** Clindamycin, Tretinoin, Derivative spectrophotometry, Pharmaceutical dosage form

## Abstract

A derivative spectrophotometric method was proposed for the simultaneous determination of clindamycin and tretinoin in pharmaceutical dosage forms. The measurement was achieved using the first and second derivative signals of clindamycin at (^1^D) 251 nm and (^2^D) 239 nm and tretinoin at (^1^D) 364 nm and (^2^D) 387 nm.

The proposed method showed excellent linearity at both first and second derivative order in the range of 60–1200 and 1.25–25 μg/ml for clindamycin phosphate and tretinoin respectively. The within-day and between-day precision and accuracy was in acceptable range (CV<3.81%, error<3.20%). Good agreement between the found and added concentrations indicates successful application of the proposed method for simultaneous determination of clindamycin and tretinoin in synthetic mixtures and pharmaceutical dosage form.

## Background

**Figure 1:**
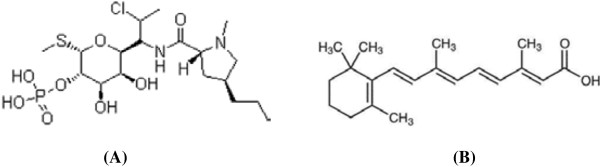
Chemical structure of A) Clindamycin phosphate and B) Tretinoin.

Clindamycin, (methyl-7-chloro-6,7,8-trideoxy-6-{[(4*R*)-1-methyl-4-propyl-L-prolyl]amino}-1-thio-L-threo-α-D-galacto-octopyranoside), (Figure 1) is a semi-synthetic derivative of lincomycin. Clindamycin reveals potent activity against many gram-positive and gram-negative bacterial infections. Topical clindamycin is used for the treatment of acne vulgaris which typically leads to suppression of cutaneous propionibacterium acnes [1].

Tretinoin, (3,7-dimethyl-9-(2,6,6-trimethyl-1-cyclohexenyl)-nona-2,4,6,8-tetraenoic acid) (Figure 1) is a derivative of vitamin A. Tretinoin could reduce the hyperkeratinization in the sebaceous follicle and accordingly decrease the sebum secretion and inflammation [1].

Acne vulgaris is a common dermatologic problem which could be treated with systemic or topical drugs. More importantly the combination therapy of topical clindamycin and tretinoin is more beneficial for the treatment of mild to moderate stages of acne vulgaris [2].

There are some reported articles in the literature for the determination of clindamycin [3-8] or tretinoin [9,10] alone or via the combination of the two drugs [11] by HPLC and gas chromatography in bulk or in pharmaceutical dosage form. HPLC method has been used in the USP35- NF30 for determination of clindamycin phosphate and tretinoin in separate gel dosage form but no simultaneous method is reported [12]. The literature survey revealed that although there were some spectrophotometric methods for the determination of clindamycin [13,14] or tretinoin [15,16] alone, no validated spectrophotometric method for simultaneous determination of clindamycin and tretinoin was reported. The spectrophotometric technique is a highly preferable method for routine analysis due to its simplicity and economical advantages. Since the spectrophotometric quantitative analysis of two or more compounds with overlapping spectra will likely prove difficult, thus the derivative spectrophotometry is a fairly useful method for analysis of a multi-compound mixture.

In this study a derivative spectrophotometric method based on zero-crossing is used for simultaneous determination of clindamycin and tretinoin.

### Experimental

#### Chemicals

Both drugs were favorably provided by Kish Medipharm Pharmaceutical Co., Kish, Iran. The purity of clindamycin and tretinoin which were used in the proposed method was 97.43 and 101.1% respectively based on the standard USP 32, NF 27 assay method.

The excipients used in the formulation of Acnomis® gel and a blank gel without active ingredients were also provided by Kish Medipharm Pharmaceutical Co. NaOH, methanol and acetonitrile were of analytical grade and purchased from Merck (Darmstadt, Germany).

The Acnomis® gel containing clindamycin phosphate (1.2 g/100 g) and tretinoin (25 mg/100 g) was also prepared by Kish Medipharm Pharmaceutical Co.

### Instruments

A double beam UV-Visible spectrophotometer (160A, Shimadzu, Japan) with a fixed 2 nm band width and 1 cm quartz cell were utilized for spectrophotometric measurement. A Waters (Milford,USA)HPLC system (A Model 510 pump, a model 717 plus auto-sampler and a model 418 UV-Visible detector) was used for HPLC determination.

### Standard solutions

Stock solutions of clindamycin phosphate and tretinoin were prepared separately in a mixture of methanol and 0.1 M NaOH (50:50) to reach a concentration of 1.2 and 250 μg/ml respectively. Synthetic mixtures of varying concentration of clindamycin phosphate (60, 120, 240, 360, 480, 720, 960 and 1200 μg/ml) in the presence of tretinoin (12.5 μg/ml) and also varying concentration of tretinoin (1.25, 2.5, 5, 7.5, 10, 15, 20 and 25 μg/ml) in the presence of clindamycin phosphate (600 μg/ml) were prepared for construction of calibration curves. The standard solutions of clindamycin and tretinoin in the synthetic mixtures for determination of relative recovery were constructed with respect to their ratio in the pharmaceutical dosage form.

### Accuracy and precision

The within-day and between-day precision and accuracy of the proposed method were calculated by analyzing three sets of synthetic mixtures of clindamycin phosphate and tretinoin in one day and three consecutive days.

### Application of the method in dosage form

A 0.5 g sample of gel formulation containing propylene glycol, butylated hydroxytoluene, simethicon, clindamycin phosphate ( 1.2 g/100) and tretinoin (25 mg/100) was weighed and transmitted to a 50 ml volumetric flask. The sample was dissolved in a mixture of methanol and 0.1 M NaOH (50:50) by sonicating for 10 min. The mixture was made up to required volume with the same solvent and centrifuged for 10 min at 4000 rpm. The clear solution was used for determination of clindamycin and tretinoin by the proposed method and also pharmacopoeial method reported for determination of clindamycin or tretinoin alone. A solution of blank gel treated by the same procedure was used as blank in spectrophotometric method.

## Results and discussion

### Selection of solvent

Tretinoin was not soluble in water. Both clindamycin and tretinoin were soluble in alcohol and chloroform but their solubility in non-aqueous solvents was lower than in methanol [17]. Their solubility was improved by adding NaOH to the methanol and consequently, a mixture of methanol and 0.1 M NaOH (50:50) was selected as a proper solvent to prepare the standard solutions.

### Absorption spectra

The zero-order spectra of clindamycin (600 μg/ml) and tretinoin (12.5 μg/ml) solutions were separately measured at 200–500 nm using the solvent (methanol: 0.1 M NaOH 50:50) as a blank.

**Figure 2:**
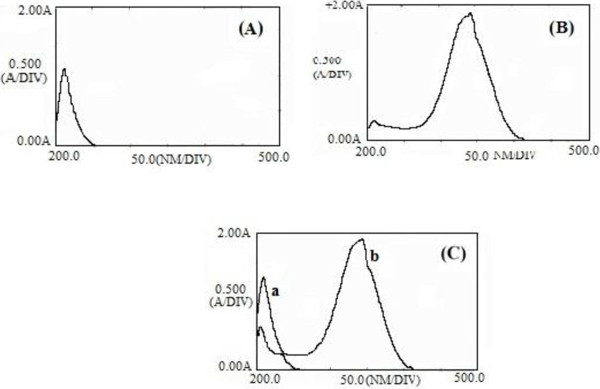
Zero-order spectra of clindamycin phosphate (A), tretinoin (B) and overlapped spectra (C).

To give a clear picture, the absorption spectra of clindamycin phosphate and tretinoin are shown in Figure 2 where the zero-order spectra demonstrated a marked overlapping. As a result, simultaneous determination of two drugs will not be possible by direct measurement of absorbance signals. The first to fourth order derivative spectra of those solutions were obtained in the same range at different Δλ values.

Zero-crossing points were assigned from the first to fourth order derivative spectra of clindamycin and tretinoin. The suitable zero-crossing points were selected based on the best linear response to the clindamycin concentration in the presence of tretinoin or the tretinoin concentration in the presence of clindamycin.

### Selection of suitable wavelengths

**Figure 3:**
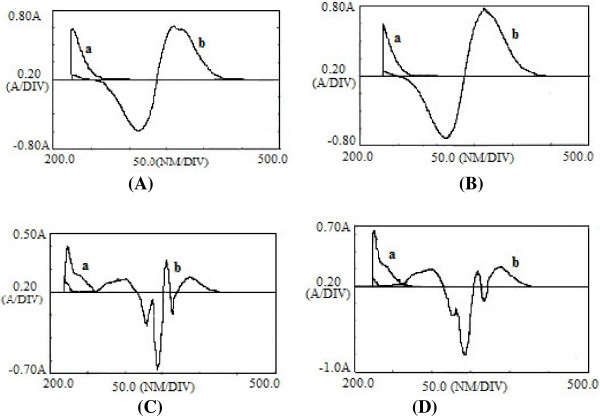
Derivative spectra of clindamycin phosphate (a) and tretinoin (b); (A) First order spectra (Δλ=16); (B) First order spectra (Δλ=20); (C) Second order spectra (Δλ=10.5); (D) second order spectra (Δλ=14).

As it is shown in Figure 3, the first and second order spectra of clindamycin and tretinoin revealed zero-crossing points for their simultaneous determination. The zero-crossing points of clindamycin and tretinoin in the first order derivative were found to be 252 (Δλ=16), 251(Δλ=20) and 364 nm (Δλ=16) and in the second order derivative were 234(Δλ=10.5), 239 nm (Δλ=14) and 386(Δλ=10.5), 387 nm (Δλ=14) respectively.

### Method validation

The validation was done according to ICH recommendations for linearity, range, accuracy and precision, limit of detection (LOD), limit of quantification (LOQ) robustness and relative recovery [18].

### Linearity

To verify the validation of Beer’s law, six series of calibration curves were plotted using the first and second order spectra in the range of 60–1200 μg/ml and 1.25–25 μg/ml for clindamycin and tretinoin respectively.

The results of calibration curves in four different Δλ and wavelengths are given in Table 1 and 2. These data illustrate that the first and second derivative spectra measurements with Δλ = 16, 20, 10.5 and 14 can be meaningfully usable. The correlation coefficient of the calibration curves (n=6) for both drugs in all wavelengths was greater than 0.998%.

**Table 1:** Statistical data of calibration curves of clindamycin in the presence of tretinoin (12.5 μg/ml)

**Parameters**	^**1**^D^*^(Δλ=16) (λ=252 nm)	^**1**^**D (Δλ=20) (λ=251 nm)**	^**2**^D^**^(Δλ=10.5) (λ=234 nm)	^**2**^**D (Δλ=14) (λ=239 nm)**
**Linearity range**	60–1200 μg/ml	60–1200 μg/ml	60–1200 μg/ml	60–1200 μg/ml
**Regression equation**	Y= 0.000120X-9.761×10^-5^	Y= 0.000185X-0.00098	Y= 0.00024X-3.912×10^-5^	Y= 0.0004282X- 0.038
**SD of slope**	1.41×10^-6^	1.63×10^-7^	1.60×10^-6^	7.80×10^-6^
**RSD of slope (%)**	0.170	0.088	0.65	1.82
**SD of intercept**	0.000839	0.000210	0.003403	0.005001
**Correlation coefficient**	0.999	0.999	0.998	0.998

* First derivative order.

**Second derivative order.

**Table 2:** Statistical data of calibration curves of tretinoin in the presence of clindamycin (600 μg/ml)

**Parameters**	^**1**^D^*^(Δλ=16) (λ=364 nm)	^**1**^**D (Δλ=20) (λ=364 nm)**	^**2**^D^**^(Δλ=10.5) (λ=386 nm)	^**2**^**D (Δλ=14) (λ=387 nm)**
**Linearity range**	1.25–25 μg/ml	1.25–25 μg/ml	1.25–25 μg/ml	1.25–25 μg/ml
**Regression equation**	Y= 0.05086X-0.00035	Y= 0.06260X-0.0024	Y= 0.010585X+ 0.00035	Y= 0.018587X+ 0.00437
**SD of slope**	3.44×10^-5^	7.12×10^-5^	1.38×10^-4^	1.38×10^-4^
**RSD of slope (%)**	0.067	0.113	1.300	0.740
**SD of intercept**	0.000774	0.000736	0.00146	0.00259
**Correlation coefficient**	0.999	0.999	0.999	0.998

* First derivative order.

**Second derivative order.

The high values of correlation coefficient and the values of y-intercepts close to zero indicate the good linearity of the calibrations.

### Accuracy and precision

In order to study the accuracy and precision, the proposed method was applied for simultaneous determination of varying concentrations of clindamycin (60, 480 and 1200 μg/ml) in the presence of tretinoin (12.5 μg/ml) and varying concentrations of tretinoin (1.25, 5 and 15 μg/ml) in the presence of clindamycin (600 μg/ml). The within-day and between-day precision and accuracy were calculated (Tables 3 and 4).

**Table 3:** Accuracy and precision data for determination of clindamycin in the presence of tretinoin (12.5 μg/ml) by different order derivative spectrophotometry

**Added (μg/ml)**	**Within-day (n = 3)**			**Between-day (n = 9)**		
	**Found (μg/ml)**	**CV (%)**	**Error (%)**	**Found (μg/ml)**	**CV (%)**	**Error (%)**
^**1**^D^*^(Δλ=16)						
**60.00**	60.50±1.58	2.62	0.83	59.96±1.76	2.94	−0.07
**480.00**	474.31±9.72	2.05	−1.18	479.44±7.89	1.64	−0.11
**1200.00**	1205.56±1.73	0.14	0.46	1201.55±4.13	0.34	0.13
^**1**^**D (Δλ=20)**						
**60.00**	60.20±0.64	1.07	0.33	59.10±1.09	1.85	−1.49
**480.00**	482.20±1.30	0.26	0.46	480.99±2.40	0.50	0.20
**1200.00**	1203.68±2.12	0.18	0.30	1205.51±2.18	0.18	0.46
^**2**^D^**^(Δλ=10.5)						
**60.00**	60.81±2.20	3.61	1.35	59.91±1.72	2.88	−0.14
**480.00**	480.06±16.34	3.40	0.01	471.36±18.00	3.81	−1.80
**1200.00**	1201.46±6.50	0.54	0.12	1201.87±9.63	0.80	0.15
^**2**^**D (Δλ=14)**						
**60.00**	60.23±1.40	2.32	0.38	61.36±1.80	2.93	2.26
**480.00**	467.14±3.70	0.80	−2.67	465.89±4.06	0.87	−2.94
**1200.00**	1215.98±18.00	1.48	1.33	1224.42±11.96	0.97	2.03

* First derivative order.

**Second derivative order.

**Table 4:** Accuracy and precision data for determination of tretinoin in the presence of clindamycin (600 μg/ml) by different order derivative spectrophotometry

**Added (μg/ml)**	**Within-day (n = 3)**			**Between-day (n = 9)**		
	**Found (μg/ml)**	**CV (%)**	**Error (%)**	**Found (μg/ml)**	**CV (%)**	**Error (%)**
^**1**^D^*^(Δλ=16)						
**1.25**	1.22±0.02	1.64	−2.37	1.21±0.02	1.63	−3.2
**5.00**	4.88±0.004	0.09	−2.38	4.88±0.01	0.21	−2.38
**15.00**	14.98±0.081	0.54	−0.13	14.95±0.05	0.34	−0.33
^**1**^**D (Δλ=20)**						
**1.25**	1.27±0.008	0.64	1.60	1.26±0.01	1.02	0.80
**5.00**	4.85±0.018	0.37	−2.94	4.84±0.02	0.41	−3.20
**15.00**	15.02±0.036	0.24	0.13	15.00±0.03	0.21	0.00
^**2**^D^**^(Δλ=10.5)						
**1.25**	1.23±0.02	1.61	−1.60	1.23±0.03	2.44	−1.60
**5.00**	4.86±0.062	1.28	−2.8	4.89±0.08	1.64	−2.23
**15.00**	15.35±0.11	0.72	2.33	15.39±0.16	1.07	2.64
^**2**^**D (Δλ=14)**						
**1.25**	1.22±0.002	0.16	−2.40	1.21±0.02	1.65	−3.20
**5.00**	4.97±0.005	0.11	−0.60	4.88±0.08	1.67	−2.37
**15.00**	15.29±0.40	2.62	1.93	15.39±0.49	3.18	2.63

* First derivative order.

**Second derivative order.

The percentage of coefficients of variations obtained was in the range of 0.09–3.81 and the percent of error was lower than 3.2% for both drugs in all the three selected concentrations which indicate good accuracy and precision of the method.

### Limit of detection & limit of quantification

The limit of detection measured based on the standard deviation of the response and slope, was in the range of 3.4-42.5 μg/ml and 0.05–0.42 μg/ml in the first and second derivative order for clindamycin phosphate and tretinoin respectively. The quantitation limit was 60 μg/ml for clindamycin phosphate and 1.25 μg/ml for tretinoin.

### Robustness

The robustness of the proposed method was assessed by changes in the ratio of methanol and NaOH and also the molarity of NaOH up to 10 percent. There was no significant difference between the results.

### Relative recovery

The recovery was determined using standard addition method. The recoveries in different derivative conditions ranged from 94.3 to 98.3% for clindamycin and 95.2–99.3% for tretinoin (Table 5).

**Table 5:** Relative recovery of clindamycin (1.2 g) and tretinoin (25 mg)

**Derivative order**	**Recovery (%) (n=4)**	
	**Tretinoin**	**Clindamycin**
^1^D^*^ (Δλ =16)	99.3 ± 1.6	98.3 ± 1.3
^1^D (Δλ =20)	98.5 ± 2.6	97.3 ± 2.6
^2^D^**^ (Δλ =10.5)	98.6 ± 1.4	95.6 ± 1.9
^2^D (Δλ =14)	95.2 ± 4.9	94.3 ± 0.5

* First derivative order.

**Second derivative order.

### Application

The proposed method was applied successfully for the analysis of clindamycin and tretinoin in pharmaceutical dosage form (Acnomis® gel) containing 1.2 g clindamycin and 25 mg tretinoin (Table 6). The results obtained from the first and second derivative order indicate the percentage of recovery to be between 94.3-99.3 in all evaluations. This technique of analysis was compared with a HPLC method [11]. Using the two-tailed t-test method it was revealed that there was no significant difference between the results obtained from these two methods (p-value>0.05).

**Table 6:** Assay results of containing Clindamycin (1.2 g) and Tretinoin (25 mg) using derivative spectrophotometry and HPLC method

**Derivative order**		**Clindamycin (g/100)**		**Tretinoin (mg/100)**	
		**Mean± SD**	**(%) Recovery**	**Mean± SD**	**(%) Recovery**
**UV/VIS**	^1^D^*^(Δλ=16)	1.16± 0.03	96.66	24.54± 0.03	98.16
	^1^D **(Δλ=20)**	1.15± 0.06	95.83	24.85± 0.03	99.40
	^2^D^**^(Δλ=10.5)	1.14±0.03	95.00	24.11± 0.02	96.44
	^2^D **(Δλ=14)**	1.16± 0.02	96.67	23.95± 0.06	95.80
**HPLC**		1.15±0.07	95.83	24.62±0.97	98.49

* First derivative order.

**Second derivative order.

## Conclusion

It was concluded that the first and second order derivative UV spectrophotometric method at Δλ= 16, 20, 10.5 and 14 could be used for simultaneous determination of clindamycin phosphate and tretinoin in their combined pharmaceutical products. This method could be used for rapid analysis of active ingredients in process and for quality control samples.

## Competing interest

The authors declare that they have no competing interest.

## Authors’ contributions

MBT has guided the project and prepared the manuscript. MN carried out the analysis and method development. SFV prepared sample and standard solutions and collaborate in analysis. ES made substantial contributions for study design, data interpretation and involved in drafting the manuscript. All authors read and approved the final manuscript.
